# Comparison of combination therapies in the management of locally advanced pancreatic cancer: Induction chemotherapy followed by irreversible electroporation vs radiofrequency ablation

**DOI:** 10.1002/cam4.3119

**Published:** 2020-05-15

**Authors:** Chaobin He, Jun Wang, Yu Zhang, Zhiyuan Cai, Xiaojun Lin, Shengping Li

**Affiliations:** ^1^ Department of Pancreatobiliary Surgery State Key Laboratory of Oncology in South China Collaborative Innovation Center for Cancer Medicine Sun Yat‐sen University Cancer Center Guangzhou China; ^2^ State Key Laboratory of Ophthalmology Zhongshan Ophthalmic Center Sun Yat‐sen University Guangzhou P.R. China

**Keywords:** chemotherapy, irreversible electroporation, locally advanced pancreatic cancer, prognosis, radiofrequency ablation

## Abstract

**Background:**

Locally advanced pancreatic cancer (LAPC) remains a challenge for current treatments. Local destructive therapies, such as irreversible electroporation (IRE) and radiofrequency ablation (RFA), were used more and more frequently in the treatment of LAPC.

**Objective:**

This study aimed to compare the efficacy of IRE with RFA in patients with LAPC.

**Methods:**

From August 2015 to August 2017, 58 LAPC patients after IRE or RFA therapy, which was performed through open approach, were retrospectively reviewed. The survival outcomes after IRE (36 patients) and RFA (18 patients) were compared after propensity score matching (PSM) analysis.

**Results:**

Before PSM analysis, IRE after the induction chemotherapy resulted in significant higher overall survival (OS) rates and progression‐free survival (PFS) rates to RFA (2‐year OS, 53.5% vs 30.8%, *P* = .013; 2‐year PFS, 28.4% vs 12.1%, *P* = .043). After PSM analysis, compared with RFA, the survival benefit of IRE was even more obvious, (2‐year OS, 53.5% vs 27.0%, *P* = .010; 2‐year PFS, 28.4% vs 6.4%, *P* = .018). For patients with tumor larger than 4 cm, IRE resulted in comparable OS and PFS between RFA and IRE while IRE also achieved better long‐term OS to RFA for those with tumor smaller than 4 cm. Multivariate analysis illustrated that IRE was a favorable prognostic factor in terms of OS and PFS in patients with LAPC.

**Conclusions:**

IRE after induction chemotherapy is superior to RFA after induction chemotherapy for treating LAPC patients while these two therapies have comparable efficacy for tumors which were larger than 4 cm.

## Graphical abstract

Locally advanced pancreatic cancer (LAPC) is a devastating disease while the standard chemotherapy remains an unmet need in the management of LAPC. Only a multidisciplinary approach can be effective in obtaining both a local tumor reduction and a systemic control of disease. Local destructive therapies were important components of the multidisciplinary treatment. Radiofrequency ablation (RFA) and irreversible electroporation (IRE) have been proposed as new treatment options in the multimodal treatment of the LAPC. Until recently, there is a lack of studies comparing treatment effects between IRE and RFA. Therefore, we aimed to investigate the effect of IRE versus RFA after the induction chemotherapy on long‐term OS and progression‐free survival (PFS) in patents with LAPC. It was the first time to show that IRE resulted in better OS and PFS than RFA after the induction chemotherapy in patients with LAPC and should be considered as the first‐line ablation modality. A new ablative method, RFA ablation followed by tumor margin accentuation by IRE, is also considerable.

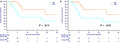

## 1 INTRODUCTION

Pancreatic cancer (PC) is associated with poor survival with a dismal 5‐year survival rate of only 7%.^1^ There was little significant progress in the treatment of PC during the past two decades.^2^, ^3^ Although surgery provides the best chance to obtain better survival, only 15% of patients were eligible candidates for surgery. More than half (55%) of patients have metastatic PC. Another 40% of patients were classified as locally advanced PC (LAPC), which were characterized with vascular involvement prohibiting upfront resection.^4^, ^5^, ^6^ There was no consensus on the most suitable treatment for patients with LAPC. The most frequently recommended treatment was chemotherapy and chemoradiotherapy, which only achieved modest survival benefit for patients with LAPC.^7^ The median overall survival (OS) was only 9‐12 months for LAPC patients treated with chemotherapy or chemoradiotherapy.^8^, ^9^, ^10^ In addition, it was shown that locally destructive disease was responsible for half of mortalities in patients with LAPC, although distant metastasis was found to be the most common form of disease progression,^11^ indicating the importance of local destructive therapies. Considering the limited success of current therapy for the local control of disease and prolonging survival of patients with LAPC, novel local destructive therapies have been tried and viewed as more and more important treatments.^12^

Nowadays, new insights have been focused on some novel local therapies as new treatment options for LAPC, including radiofrequency ablation (RFA) and irreversible electroporation (IRE).^13^ Radiofrequency ablation has been applied in solid organ malignancies, such as renal carcinoma,^14^ hepatocellular carcinoma,^15^ and LAPC.^16^, ^17^, ^18^ Also, as a subsequent treatment after induction chemotherapy in LAPC, there were many studies illustrating the survival benefit of IRE.^19^, ^20^, ^21^ As a nonthermal method, IRE creates defects in cell membrane through the transmission of high‐voltage currents through the tumor, inducing loss of homeostasis and apoptotic death of tumor cells.^21^ However, there is only limited evidence of which ablation method is survival beneficial to the LAPC patients.^22^ Therefore, the primary aim of this study was the OS comparison and the secondary aim was the progression‐free survival (PFS) comparison in LAPC patients who received IRE and RFA after the induction chemotherapy.

## 2 METHODS

### 2.1 Patients

Patients who were diagnosed with LAPC and had received IRE or RFA combined with induction chemotherapy from August 2015 to August 2017 at Sun Yat‐sen University Cancer Center were retrospectively reviewed. The diagnosis of LAPC and the final therapy were confirmed by a multidisciplinary team, which included specialized pancreatic surgeons, oncologists, pathologists, and radiologists. Patients who were pathologically confirmed pancreatic adenocarcinoma and radiologically confirmed LAPC were included into this study. Locally advanced pancreatic cancer was defined as the description of AJCC staging system for pancreatic cancer.^23^, ^24^ All LAPC patients had received four months of induction chemotherapy (FOLFIRINOX or Gem‐based chemotherapy)^25^ and those who were also judged as unresectable ones after induction chemotherapy were included in this study. A total of 378 patients were included into this study and 303 patients were excluded based on the following exclusion criteria: (a) second primary cancer; (b) distant metastases; (c) other treatments, such as surgical resection and radiotherapy; (d) a history of heart arrhythmia; and (e) missing or incomplete information. Finally, 75 patients were enrolled into this study. The study protocol conformed to the ethical guidelines of the 1975 Declaration of Helsinki and obtained approval from the Ethics Committee of Sun Yat‐sen University Cancer Center. Written informed consent was obtained from all patients.

### 2.2 Data and treatment procedure

The associated clinical data were retrieved and analyzed. Carbohydrate antigen 19‐9 (CA19‐9) and carcinoembryonic antigen (CEA) were taken after chemotherapy and prior to ablation. Before induction chemotherapy, which was conducted as the procedures described in our previous study,^25^ biopsy was finished for all patients and tumor grade was determined. After induction chemotherapy, as long as no metastases were detected, IRE or RFA was performed and the same line of chemotherapy was followed after IRE or RFA therapy. A diagnostic laparoscopy is adopted to confirm that no metastasis is present. If none is found, IRE or RFA is performed. As shown in our previous study,^25^ specialized pancreatic surgeons performed all IRE and RFA, which were performed using an open technique and guided by intraoperative ultrasound. The general anesthesia with deep neuromuscular block was adopted. In the procedure of IRE, under the guidance of ultrasound during surgery, 2 to 6 probes were adopted to create an electric field around the tumor, which caused nanoscale pore formation in the cell membrane. Also, the electrode of RFA was placed at the center of tumor. The ablation of IRE and RFA was monitored with ultrasound during surgery. The same line of chemotherapy was performed 7‐14 days after IRE treatment. According to the guidelines from National Comprehensive Cancer Network (NCCN),^5^ 4 cm was adopted as the cutoff value of tumor size in this study. Tumors which were larger than 4 cm were classified as large ones for pancreatic cancer. To further compare the efficacy of IRE and RFA in LAPC patients with large tumors, the survival comparisons of IRE and RFA were conducted in patients with tumors that are larger than 4 cm.

### 2.3 Follow‐up

The follow‐up procedures, including hematological examination, such as CA19‐9 and CEA analysis, and radiological examination (abdominal CT or MRI) were regularly performed for patients, who had the first one at approximately 1 month after IRE or RFA and the following ones every 2‐3 months thereafter. OS and PFS were defined as the duration from the date of induction chemotherapy until death or disease progression. If no endpoint event was observed, the date of last follow‐up was also used to calculate OS or PFS. The last follow‐up was completed on September 30, 2018.

### 2.4 Propensity score matching (PSM) analysis

To minimize selection bias, PSM analysis was utilized based on the following factors: age, gender, tumor site, tumor size, tumor grade, TNM stage, CA19‐9, and CEA. A two‐to‐one nearest‐neighbor matching algorithm^26^ and “MatchIt” package in R software were adopted to perform PSM analysis.

### 2.5 Statistical analysis

The independent sample *t* test, Mann‐Whitney *U* test, and chi‐square test were used to compare the continuous and categorical variables, respectively. The survival differences in terms of OS and PFS were compared by the log‐rank test and survival curves were analyzed using the Kaplan‐Meier method. Prognostic factors of survival and the associated corresponding 95% confidence intervals (CIs) were determined by multivariate analyses using the Cox regression model. Statistical significance was considered when two‐tailed *P* value < .05 was obtained. All statistical analyses were performed using R software version 3.4.2 software (R Foundation for Statistical Computing, Vienna, Austria).

## 3 RESULTS

### 3.1 Patient

A total of 75 consecutive LAPC patients were identified to have IRE or RFA after the induction chemotherapy. Among this cohort, 8 patients were excluded due to other treatments other than IRE or RFA. Additional patients were excluded due to metastatic diseases developed after the induction chemotherapy (n = 5), a history of second primary malignant tumors (n = 3), or a history of heart arrhythmia (n = 1). After the exclusion process, there were 58 patients available for analysis (IRE: 36 and RFA: 22). The baseline characteristics of patients allocated to IRE or RFA were described in Table 1. Patients in the IRE group were likely to have tumors located in the head of pancreas while tumors with tumor‐node‐metastasis (TNM) stage III was a little more frequently observed in patients in this group. After PSM analyses, 36 patients in the IRE group and 18 patients in the RFA group were matched and compared. FOLFIRINOX‐ and Gem‐based chemotherapy were applied to 21 (58.3%) and 15 (41.7%) patients in the IRE group, which was similar with that of the RFA group. All other factors were balanced between two groups after PSM analysis.

**Table 1:** Comparisons of clinical and imaging characteristics of patients

Characteristic		Before PSM				After PSM			
		Chemotherapy + IRE n (%)	Chemotherapy + RFA n (%)	Total number	*P* value	Chemotherapy + IRE n (%)	Chemotherapy + RFA n (%)	Total number	*P* value
Total number		36 (62.1)	22 (37.9)	58		36 (66.7)	18 (33.3)	54	
Age (y)^a^	≤60	20 (69.0)	9 (31.0)	29	.417	20 (74.1)	7 (25.9)	27	.387
	>60	16 (55.2)	13 (44.8)	29		16 (59.3)	11 (40.7)	27	
Gender^a^	Female	19 (67.9)	9 (32.1)	28	.427	19 (67.9)	9 (32.1)	28	.847
	Male	17 (56.7)	13 (43.3)	30		17 (65.4)	9 (34.6)	26	
Tumor size^a^ (cm)	≤2	1 (100.0)	0 (0.0)	1	.505	1 (100.0)	0 (0.0)	1	.685
	2 ~ 4	20 (66.7)	10 (33.3)	30		20 (69.0)	9 (31.0)	29	
	>4	15 (55.6)	12 (44.4)	27		15 (62.5)	9 (37.5)	24	
Tumor grade^a^	Well	3 (60.0)	2 (40.0)	5	.994	3 (60.0)	2 (40.0)	5	.908
	Moderate	20 (62.5)	12 (37.5)	32		20 (69.0)	9 (31.0)	29	
	Poor	13 (61.9)	8 (38.1)	21		13 (65.0)	7 (35.0)	20	
LN metastasis	Absent	9 (52.9)	8 (47.1)	17	.387	9 (60.0)	6 (40.0)	15	.536
	Present	27 (65.9)	14 (34.1)	41		27 (69.2)	12 (30.8)	39	
Tumor site^a^	Head	18 (64.3)	10 (35.7)	28	.018	18 (64.3)	10 (35.7)	28	.207
	Body	15 (78.9)	4 (21.1)	19		15 (78.9)	4 (21.1)	19	
	Tail	3 (27.3)	8 (72.7)	11		3 (42.9)	4 (57.1)	7	
TNM stage^a^	IIB	4 (30.8)	9 (69.2)	13	.020	4 (40.0)	6 (60.0)	10	.067
	III	32 (71.1)	13 (28.9)	45		32 (72.7)	12 (27.3)	44	
WBC (*10^9^)	≤10	32 (61.5)	20 (38.5)	52	.589	32 (66.7)	16 (33.3)	48	.687
	>10	4 (66.7)	2 (33.3)	6		4 (66.7)	2 (33.3)	6	
HGB (g/L)	≤120	1 (50.0)	1 (50.0)	2	.619	1 (50.0)	1 (50.0)	2	.560
	>120	35 (62.5)	21 (37.5)	56		35 (67.3)	17 (32.7)	52	
PLT (*10^9^)	≤300	31 (60.8)	20 (39.2)	51	.698	31 (64.6)	17 (35.4)	48	.651
	>300	5 (71.4)	2 (28.6)	7		5 (83.3)	1 (16.7)	6	
ALT (U/L)	≤40	26 (66.7)	13 (33.3)	39	.390	26 (70.3)	11 (29.7)	37	.536
	> 40	10 (52.6)	9 (47.4)	19		10 (58.8)	7 (41.2)	17	
AST (U/L)	≤ 40	29 (63.0)	17 (37.0)	46	.752	29 (67.4)	14 (32.6)	43	.537
	>40	7 (58.3)	5 (41.7)	12		7 (63.6)	4 (36.4)	11	
ALP (U/L)	≤100	19 (61.3)	12 (38.7)	31	.556	19 (65.5)	10 (34.5)	29	.539
	>100	17 (63.0)	10 (37.0)	27		17 (68.0)	18 (72.0)	25	
GGT (U/L)	≤45	19 (70.4)	8 (29.6)	27	.283	19 (73.1)	7 (26.9)	26	.395
	>45	17 (54.8)	14 (45.2)	31		17 (60.7)	11 (39.3)	28	
ALB (g/L)	≤ 40	4 (44.4)	5 (55.6)	9	.278	4 (50.0)	4 (50.0)	8	.418
	>40	32 (65.3)	17 (34.7)	49		32 (69.6)	14 (30.4)	46	
TBIL (µmol/L)	≤20.5	27 (61.4)	17 (38.6)	44	.553	27 (67.5)	13 (32.5)	40	.536
	>20.5	9 (64.3)	5 (35.7)	14		9 (64.3)	5 (35.7)	14	
IBIL (µmol/L)	≤15	32 (62.7)	19 (37.3)	51	.540	32 (68.1)	15 (31.9)	47	.674
	>15	4 (57.1)	3 (42.9)	7		4 (57.1)	3 (42.9)	7	
CRP (ng/L)	≤3	24 (64.9)	13 (35.1)	37	.585	24 (64.9)	13 (35.1)	37	.763
	>3	12 (57.1)	9 (42.9)	21		12 (70.6)	5 (29.4)	17	
CEA (ng/mL)^a^	≤5	21 (70.0)	9 (30.0)	30	.250	21 (72.4)	8 (27.6)	29	.378
	>5	15 (53.6)	13 (46.4)	28		15 (60.0)	10 (40.0)	25	
CA19‐9 (U/mL)^a^	≤35	9 (60.0)	6 (40.0)	15	.501	9 (64.3)	5 (35.7)	14	.536
	>35	27 (62.8)	16 (37.2)	43		27 (67.5)	13 (32.5)	40	
HBsAg	Negative	33 (62.3)	20 (37.7)	53	.635	33 (66.0)	17 (34.0)	50	.593
	Positive	3 (60.0)	2 (40.0)	5		3 (75.0)	1 (25.0)	4	
Chemotherapy	FOLFIRINOX	21 (67.7)	10 (32.3)	31	.420	21 (72.4)	8 (27.6)	29	.394
	Gem	15 (55.6)	12 (44.4)	27		15 (60.0)	10 (40.0)	25	

Abbreviations: ALB, albumin; ALP, alkaline phosphatase; ALT, alanine transaminase; AST, aspartate aminotransferase; CA19‐9, carbohydrate antigen 19‐9; CEA, carcinoembryonic antigen; CRP, C‐reactive protein; GGT, glutamyl transpeptidase; HBsAg, hepatitis B surface antigen; HBsAg, hepatitis B surface antigen; IBIL, indirect bilirubin; LN, lymph node metastasis; PLT, platelet count; TBIL, total bilirubin; TNM, tumor‐node‐metastasis stage; WBC, white blood cell count.

^a^ Variables that were used for propensity score matching analysis.

### 3.2 Survival and tumor progression analysis

**Figure 1:**
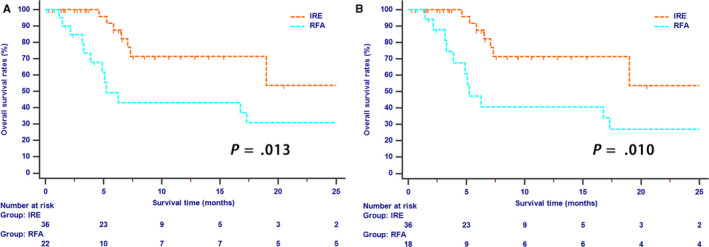
The Kaplan‐Meier survival curves of overall survival stratified by treatment strategies for patients with LAPC before (A) and after (B) propensity score matching

The whole study cohort was regularly followed up at a median time of 10.0 months (range 1.2‐75.0 months). The 1‐ and 2‐year OS rates were 60.7% and 42.5%, respectively. A total of 7 (19.4%) deaths and 11 (61.1%) deaths were observed in the IRE and RFA groups, respectively (*P* = .005). Before PSM analysis, the 1‐ and 2‐year OS rates for patients in the IRE and RFA groups were 71.4%, 53.5% and 41.3%, 30.8%, respectively (*P* = .013, Figure 1A). After PSM analysis, patients in the IRE group still had significant higher OS rates than those in the RFA group (1‐year OS, 71.4% vs 40.5%; 2‐year OS, 53.5% vs 27.0%; *P* = .010, Figure 1B).

**Figure 2:**
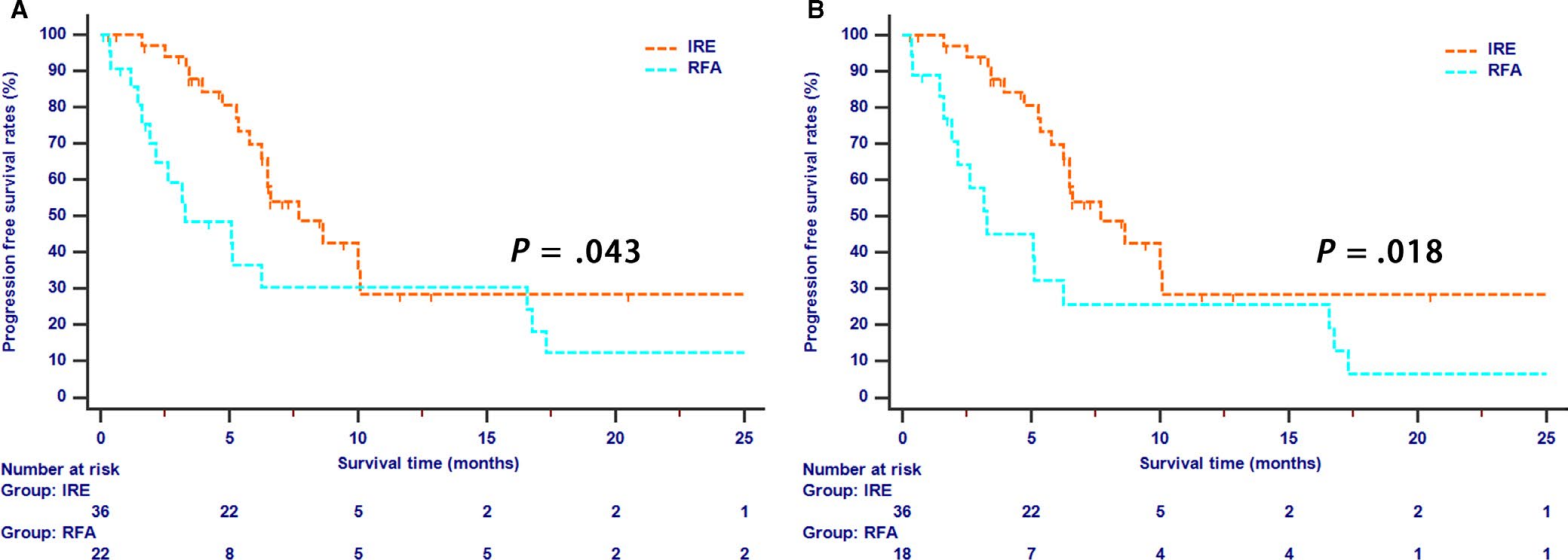
The Kaplan‐Meier survival curves of progression‐free survival stratified by treatment strategies for patients with LAPC before (A) and after (B) propensity score matching

Tumor progression was recorded in 17 (47.2%) and 15 (83.3%) patients in the IRE and RFA groups, respectively (*P* = .018). Local recurrences were observed in 6 (16.6%) patients in the IRE group and 5 (27.7%) patients in the RFA group. In terms of distant metastases, patients in the RFA group have more cases (n = 10, 55.5%) than those in IRE group (n = 11, 30.5%). The 1‐ and 2‐year PFS rates for patients in the IRE and RFA groups were 28.4% and 28.4%, and 30.3% and 12.1%, respectively (*P* = .043, Figure 2A) before PSM analysis while after PSM analysis, 1‐ and 2‐year PFS rates for patients in the IRE and RFA groups were 28.4% and 28.4%, and 25.7% and 6.4%, respectively (*P* = .018, Figure 2B).

**Figure 3:**
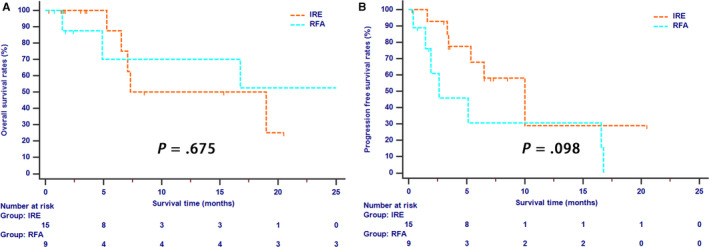
The Kaplan‐Meier survival curves of overall survival (A) and progression‐free survival (B) stratified by treatment strategies for LAPC patients whose tumor was larger than 4 cm

**Figure 4:**
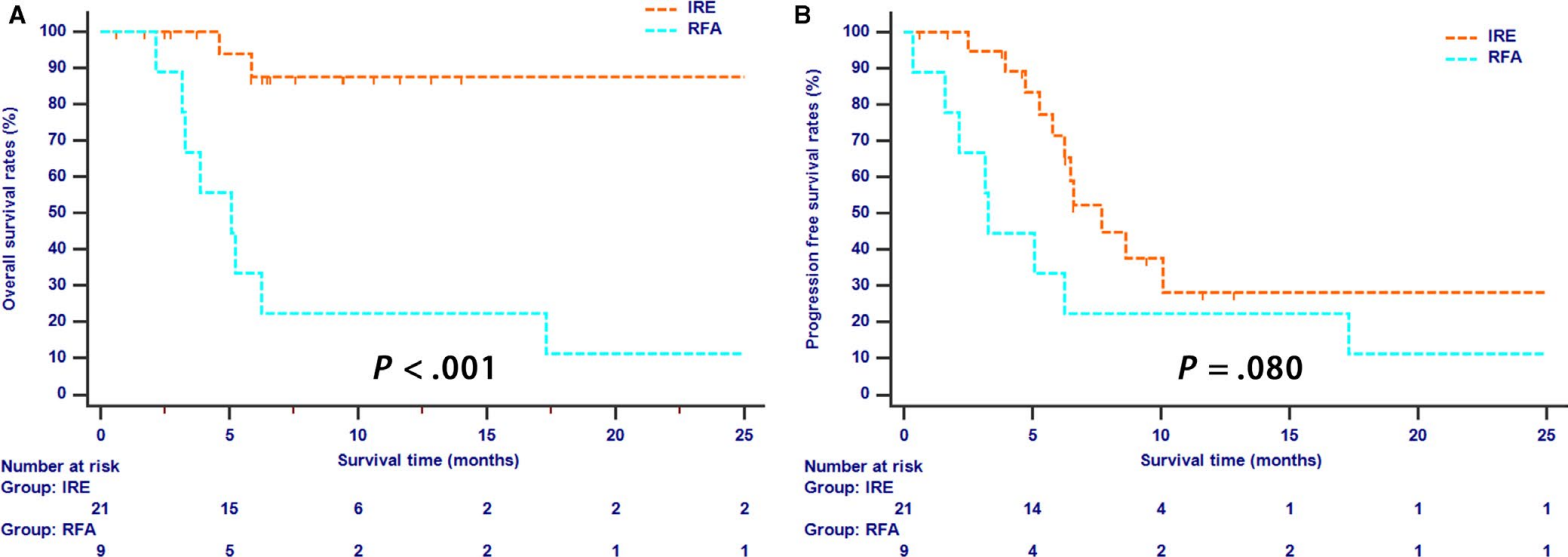
The Kaplan‐Meier survival curves of overall survival (A) and progression‐free survival (B) stratified by treatment strategies for LAPC patients whose tumor was smaller than 4 cm

Tumor size was an important factor which may have an effect on the efficacy of ablation therapy. Four centimeter was adopted as the cutoff value of tumor size in this study. Fifteen (41.7%) patients and 9 (50%) patients had tumors which were larger than 4 cm in the IRE and RFA groups, respectively. For cases with LAPC larger than 4 cm, long‐term OS (*P* = .675, Figure 3A) and PFS (*P* = .098, Figure 3B) rates were similar between two groups. However, patients whose tumor sizes were smaller than 4 cm had significantly higher OS rates in the IRE group than those in the RFA group (*P* < .001, Figure 4A). In addition, the survival benefit for PFS were also different between two groups, although the difference was not significant (*P* = .080, Figure 4B).

### 3.3 Prognostic factors associated with OS and PFS

As shown in Table 2, univariate analysis revealed that IRE treatment, tumor grade, ALB, and CRP were associated with OS. Moreover, multivariate analysis identified several independent prognostic factors, including chemotherapy followed by IRE treatment (HR = 4.120; 95% CI, 1.493‐11.371; *P* = .006) and ALB level (HR = 0.240, 95% CI, 0.074‐0.780, *P* = .018). For PFS analysis, the only factor identified by univariate and multivariate analyses was IRE treatment (IRE vs RFA, HR = 2.330; 95% CI, 1.138‐4.768; *P* = .021) (Table 3).

**Table 2:** Univariate and multivariate analyses of OS in patients

Characteristic		Before PSM						After PSM					
		Univariate analysis			Multivariate analysis			Univariate analysis			Multivariate analysis		
		HR	95%CI	*P*	HR	95%CI	*P*	HR	95%CI	*P*	HR	95% CI	*P*
Age (y)	≤60/ >60	1.408	0.565‐3.508	.462			NI	1.155	0.455‐2.933	.761			NI
Gender	Female/ Male	1.515	0.596‐3.851	.383			NI	1.529	0.592‐3.951	.380			NI
Tumor size (cm)	≤2/2 ~ 4/>4	0.927	0.394‐2.180	.861			NI	1.109	0.458‐2.687	.818			NI
Tumor grade	Well/ Moderate/ Poor	2.921	1.259‐6.774	.013	2.715	1.121‐6.575	.027	2.574	1.114‐5.949	.027	2.162	0.894‐5.227	.087
LN metastasis	Absent/ Present	2.551	0.741‐8.787	.138			NI	2.563	0.737‐8.916	.139			NI
Tumor site	Head/ Body/ Tail	1.151	0.629‐2.103	.649			NI	1.170	0.608‐2.249	.638			NI
WBC (*10^9^)	≤10/ >10	1.642	0.477‐5.651	.432			NI	1.662	0.480‐5.761	.423			NI
HGB (g/L)	≤120/ >120	0.556	0.073‐4.221	.570			NI	0.540	0.071‐4.119	.552			NI
PLT (*10^9^)	≤300/ >300	0.898	0.260‐3.105	.865			NI	1.269	0.357‐4.512	.712			NI
ALT (U/L)	≤40/ >40	0.760	0.287‐2.016	.581			NI	0.697	0.247‐1.966	.495			NI
AST (U/L)	≤40/ >40	1.466	0.484‐4.440	.499			NI	1.059	0.305‐3.671	.929			NI
ALP (U/L)	≤100/ >100	0.900	0.361‐2.244	.822			NI	0.864	0.334‐2.233	.763			NI
GGT (U/L)	≤45/ >45	1.205	0.488‐2.973	.686			NI	1.218	0.482‐3.075	.676			NI
ALB (g/L)	≤40/ >40	0.167	0.060‐0.467	.001	0.189	0.062‐0.581	.004	0.200	0.068‐0.593	.004	0.240	0.074‐0.780	.018
TBIL (µmol/L)	≤20.5/ >20.5	0.661	0.192‐2.270	.511			NI	0.656	0.190‐2.268	.505			NI
IBIL (µmol/L)	≤15/ >15	1.270	0.288‐5.598	.752			NI	1.318	0.297‐5.853	.716			NI
CRP (ng/L)	≤3/ >3	2.613	1.047‐6.519	.039	1.384	0.511‐3.746	.523	3.127	1.185‐8.250	.021	1.848	0.603‐5.662	.282
CEA (ng/mL)	≤5/ >5	1.488	0.556‐3.979	.429			NI	1.674	0.626‐4.476	.304			NI
CA19‐9 (U/ml)	≤35/ >35	1.967	0.643‐6.020	.236			NI	2.103	0.684‐6.468	.195			NI
HBsAg	Negative/ Positive	0.882	0.116‐6.686	.903			NI	0.908	0.119‐6.918	.926			NI
Group	IRE/ RFA	3.151	1.220‐8.140	.018	3.870	1.426‐10.503	.008	3.320	1.264‐8.718	.015	4.120	1.493‐11.371	.006
Chemotherapy type	FOLFIRINOX/ Gem	0.812	0.508‐1.299	.385			NI	0.729	0.445‐1.192	.208			NI

Abbreviations: ALB, albumin; ALP, alkaline phosphatase; ALT, alanine transaminase; AST, aspartate aminotransferase; CA19‐9, carbohydrate antigen 19‐9; CEA, carcinoembryonic antigen; CRP, C‐reactive protein; GGT, glutamyl transpeptidase; HBsAg, hepatitis B surface antigen; HBsAg, hepatitis B surface antigen; IBIL, indirect bilirubin; LN, lymph node metastasis; PLT, platelet count; TBIL, total bilirubin; TNM, tumor‐node‐metastasis stage; WBC, white blood cell count.

**Table 3:** Univariate and multivariate analyses of PFS in patients

Characteristic		Before PSM						After PSM					
		Univariate analysis			Multivariate analysis			Univariate analysis			Multivariate analysis		
		HR	95%CI	*P*	HR	95%CI	*P*	HR	95% CI	*P*	HR	95% CI	*P*
Age (years)	≤60/ >60	1.142	0.572‐2.279	.707			NI	0.927	0.457‐1.884	.835			NI
Gender	Female/ Male	1.448	0.722‐2.902	.297			NI	1.650	0.803‐3.390	.173			NI
Tumor size (cm)	≤2/ 2 ~ 4/ >4	0.950	0.497‐1.815	.876			NI	1.141	0.587‐2.215	.697			NI
Tumor grade	Well/ Moderate/ Poor	1.232	0.691‐2.199	.480			NI	1.098	0.619‐1.950	.749			NI
LN metastasis	Absent/ Present	1.434	0.645‐3.189	.376			NI	1.444	0.646‐3.226	.370			NI
Tumor site	Head/ Body/ Tail	0.973	0.613‐1.545	.909			NI	1.077	0.653‐1.776	.771			NI
WBC (*10^9^)	≤10/ >10	1.560	0.592‐4.109	.369			NI	1.542	0.583‐4.078	.383			NI
HGB (g/L)	≤120/ >120	0.515	0.122‐2.180	.367			NI	0.520	0.122‐2.205	0.375			NI
PLT (*10^9^)	≤300/ >300	0.434	0.132‐1.427	.169			NI	0.604	0.181‐2.011	.411			NI
ALT (U/L)	≤40/ >40	0.800	0.380‐1.683	.557			NI	0.832	0.384‐1.803	.642			NI
AST (U/L)	≤40/ >40	1.275	0.553‐2.943	.569			NI	1.025	0.421‐2.495	.956			NI
ALP (U/L)	≤100/ >100	0.578	0.284‐1.175	.130			NI	0.578	0.279‐1.200	.142			NI
GGT (U/L)	≤45/ >45	0.606	0.300‐1.225	.163			NI	0.635	0.309‐1.303	.216			NI
ALB (g/L)	≤40/ >40	0.392	0.165‐0.930	.034	0.372	0.156‐0.888	0.026	0.471	0.189‐1.175	.107			NI
TBIL (µmol/L)	≤20.5/ >20.5	0.594	0.243‐1.453	.254			NI	0.544	0.220‐1.345	.187			NI
IBIL (µmol/L)	≤ 5/ >15	1.580	0.603‐4.137	.352			NI	1.556	0.592‐4.088	.370			NI
CRP (ng/L)	≤3/ >3	0.975	0.461‐2.062	.946			NI	1.120	0.512‐2.450	.777			NI
CEA (ng/mL)	≤5/ >5	1.312	0.638‐2.699	.460			NI	1.487	0.724‐3.053	.280			NI
CA19‐9 (U/ml)	≤35/ >35	1.735	0.749‐4.021	.199			NI	1.846	0.794‐4.291	.155			NI
HBsAg	Negative/ Positive	1.258	0.380‐4.164	.707			NI	1.230	0.371‐4.082	.735			NI
Group	IRE/ RFA	2.041	1.009‐4.129	.047	2.125	1.043‐4.330	0.038	2.330	1.138‐4.768	.021	2.330	1.138‐4.768	0.021
Chemotherapy type	FOLFIRINOX/ Gem	1.084	0.769‐1.530	.644			NI	1.014	0.716‐1.438	.936			NI

Abbreviations: ALB, albumin; ALP, alkaline phosphatase; ALT, alanine transaminase; AST, aspartate aminotransferase; CA19‐9, carbohydrate antigen 19‐9; CEA, carcinoembryonic antigen; CRP, C‐reactive protein; GGT, glutamyl transpeptidase; HBsAg, hepatitis B surface antigen; HBsAg, hepatitis B surface antigen; IBIL, indirect bilirubin; LN, lymph node metastasis; PLT, platelet count; TBIL, total bilirubin; TNM, tumor‐node‐metastasis stage; WBC, white blood cell count.

### 3.4 Complications comparison

As shown in Table 4. No intra‐abdominal hemorrhage after treatment was observed in both groups. Drainage of seroperitoneum was conducted in one patient in the IRE group and two patients in the RFA group. In addition, acute pancreatitis occurred in two patients in the RFA group while no cases occurred in the IRE group. In terms of minor complications, fever and pain were the most common complications in both groups. Significantly more cases in the RFA group (9 of 18 patients) had fever than those in the IRE group (4 of 36 patients) while similar proportions of patients in both groups had pain after treatment and required analgesics. Notably, more patients in the RFA group had vomiting (4 of 18 patients) than those in IRE group (1 of 36 patients). All patients with complications received appropriate therapy and reached the discharge criteria.

**Table 4:** Procedure‐related complications

Variables	Before PSM			After PSM		
	IRE (n = 36)	RFA (n = 22)	*P*	IRE (n = 36)	RFA (n = 18)	*P*
Major complications	2	7		2	7	
Seroperitoneum (require drainage)	1	2	.551	1	2	.255
Acute pancreatitis	0	2	.140	0	2	.107
Abdominal infection	1	3	.148	1	3	.103
Minor complications	24	37		24	33	
Fever (axillary temperature > 38.5℃)	4	11	.002	4	9	.005
Pain (requiring analgesics)	23	18	.234	23	16	.062
Diarrhea	2	3	.357	2	3	.319
Vomiting	1	4	.063	1	4	.038

## 4 DISCUSSION

Locally advanced pancreatic cancer, a devastating disease, owns relatively high mortalities and extremely low long‐term survival rates.^27^, ^28^ IRE and RFA can be used as local ablation methods after the induction chemotherapy for patients with LAPC, while the comparison of treatment efficacy of IRE and RFA remains unclear. The present study showed that although patients in the IRE group were associated with more advanced TNM stages, IRE was superior to RFA with respect to 1‐ and 2‐year OS and PFS for all patients and those whose tumor sizes were smaller than 4 cm. The survival differences were even more obvious when the baseline factors were balanced between two groups.

As a local thermal ablative method, RFA generates local high temperatures through the high‐frequency alternating current, leading to coagulative necrosis and protein denaturation inside neoplastic tissue.^22^ The efficacy is partly limited by the heat sink effect in the heavily vascularized pancreas. Heat was dissipated by the blood vessels near ablation probes, leading to an area of lower temperature of the neighboring tumor cells.^29^, ^30^ Moreover, during the procedure of RFA, temperatures higher than 90℃ could induce thermal injuries and the injuries will increase with the elevation of temperatures. Therefore, the whole tumor ablation usually is avoided. The procedures of pull‐backs of the tips left a “security ring” at the periphery of the tumor, preventing high temperature diffusing to healthy surrounding tissues.^29^, ^31^ Therefore, to some extent, RFA can hardly achieve complete ablation in PDAC.^32^ The incomplete ablation would result in rapid local recurrence and decreased long‐term survival. In contrast, relying on the application of short and high‐voltage current pulses through the tumor, IRE causes irreversible permeabilization in cell membrane integrity and induces subsequent apoptosis.^33^ Therefore, IRE is not affected by the heat sink effect. The use of multiple needles allows bracketing the artery and leads to negligible amount of heat. Therefore, whole tumor can be surrounded by electric field of extremely high voltage without harming nearby important structure around pancreas.^22^ Therefore, the application of IRE seems to be more appropriate than RFA for PDAC, which is characterized by encapsulating celiac axis or superior mesenteric artery. In addition, by disrupting the dense stroma of LAPC and reconstruction of microcirculation,^34^ IRE contributed to the chemotherapy delivery to tumor, which also partly explained the survival benefit of IRE combined with systemic chemotherapy in patients with LAPC. Additionally, compared to other thermal ablative methods, IRE owns the nonthermal feature, which ensures the clinical effect is free of heat sink effect and leaves the supporting tissue largely unaffected. Considering the nature of preservation of vessels which is helpful for the transmission of immune molecules or cells, IRE may be more immunological sensitive than thermal ablations. Robert et al had shown that greater immune effect and therapeutic efficacy caused by IRE therapy in immunocompetent mice were observed than those in immunodeficient models, indicating that IRE could induce a systemic response beyond the targeted ablation region.^35^ Previous studies have illustrated the immune‐stimulation effect induced by IRE was helpful for the survival elevation,^36^, ^37^, ^38^ which would also act as the reason why IRE could work better than RFA in improving survival in LAPC patients.

A summary of studies which included a total of 106 LAPC patients after RFA treatment showed that the median postoperative complication rate and mortality were 28.3% and 7.5%, respectively.^32^ The median survival was 6.5 months in that study, which was similar with that of our results. In this study, LAPC patients who were treated with IRE combined with induction chemotherapy had a median OS of 21.6 months, which was in accordance with the results from study of the largest cohort conducted by Martin et al^39^ Moreover, Martin et al also reported a median PFS of 12.4 months, which was higher than that of our study. However, radical resection and margin accentuation by IRE were applied in nearly a quarter of all patients while our study only focused on LAPC patients who were not candidates for surgical resection. Similar with other study which showed that IRE could reduce local recurrence by allowing increased drug delivery to the tissue in the reversible electroporation zone,^40^ IRE combined with chemotherapy, as a kind of multidiscipline approaches, could contribute to the elevated PFS rates. In this study, the median PFS was 7.7 months for patients in the IRE group, which was significantly longer than that of patients in the RFA group. Therefore, across‐study comparisons of long‐term survival consolidated the survival benefit of IRE over RFA for patients with LAPC. Moreover, it was reported that the complication rate and mortality of IRE were 29% and 2%, respectively,^39^, ^41^ indicating that compared with RFA, IRE was a more feasible and safe local ablation method.

In addition, the prognostic factors for patients with LAPC were explored and it was found that compared with IRE, RFA contributed to better OS and PFS. Also, low albumin level was an unfavorable prognostic factor for OS and PFS, whereas poorly differentiated tumor were negative prognostic factor for OS, consistent with results from previous studies.^42^, ^43^ Interestingly, when subgroup analyses stratified by tumor size were conducted, it was shown that patients had similar OS and PFS rates in both the IRE and RFA groups if tumor sizes were larger than 4 cm; although IRE displayed better in elevated long‐term survival rates in all patients or patients whose tumor sizes were smaller than 4 cm. Compared with a single ablation, multiple overlapping ablations may partly enlarge the possible ablation area and shrink the “security ring” around the tumor. Moreover, due to the presence of viable “security ring” of RFA at the periphery of tumor, the addition of IRE targeted at this area will make a complete ablation. Therefore, combining the advantages of IRE and RFA, it was suggested that maybe the combination of RFA ablation followed by tumor margin accentuation by IRE was a feasible local destructive method for the treatment of patients with LAPC. However, more appropriate randomized controlled studies are needed to evaluate the feasibility and efficacy of this new combination therapy.

There were several limitations which should be considered. The small sample size of patients and the potential patient selection bias kept us from drawing definitive conclusions. To improve the intergroup comparability, PSM analysis was applied to reduced selection bias. Large‐scale prospective randomized controlled studies are warranted to confirm results of this study.

In conclusion, it was the first time to show that compared with RFA, IRE resulted in better survival after the induction chemotherapy in LAPC patients and should be considered as the first‐line ablation modality. The efficacy of the combination therapy of IRE and induction chemotherapy is needed to be confirmed by randomized clinical trials.

## CONFLICT OF INTERESTS

The authors declare that they have no conflict of interest.

## AUTHORS CONTRIBUTIONS

Chaobin He and Shengping Li designed the study. Chaobin He, Jun Wang, and Yu Zhang analyzed and interpreted the data and wrote the manuscript. Zhiyuan Cai and Xiaojun Lin collected the data. Shengping Li revised the manuscript.

## Data Availability

The authenticity of this article has been validated by uploading the key raw data onto the Research Data Deposit public platform (http://www.researchdata.org.cn), with the Approval Number as RDDA2019001060.
